# Emergency Department Frequent Utilization for Non-Emergent Presentments: Results from a Regional Urban Trauma Center Study

**DOI:** 10.1371/journal.pone.0147116

**Published:** 2016-01-19

**Authors:** Joshua G. Behr, Rafael Diaz

**Affiliations:** 1 Virginia Modeling, Analysis and Simulation Center, Old Dominion University, Suffolk, Virginia, United States of America; 2 Zaragoza Logistics Center, Massachusetts Institute of Technology, Zaragoza, España; University of California San Diego, UNITED STATES

## Abstract

**Objectives:**

First, to test a model of the drivers of frequent emergency department utilization conceptualized as falling within predisposing, enabling, and need dimensions. Second, to extend the model to include social networks and service quality as predictors of frequent utilization. Third, to illustrate the variation in thresholds that define frequent utilization in terms of the number of emergency department encounters by the predictors within the model.

**Data Source:**

Primary data collection over an eight week period within a level-1 trauma urban hospital’s emergency department.

**Study Design:**

Representative randomized sample of 1,443 adult patients triaged ESI levels 4–5. Physicians and research staff interviewed patients as they received services. Relationships with the outcome variable, utilization, were tested using logistic regression to establish odds-ratios.

**Principal Findings:**

70.6 percent of patients have two or more, 48.3 percent have three or more, 25.3 percent have four or more, and 14.9 percent have five or more emergency department visits within 12 months. Factors associated with frequent utilization include gender, race, poor mental health, mental health drugs, prescription drug abuse, social networks, employment, perceptions of service quality, seriousness of condition, persistence of condition, and previous hospital admittance.

**Conclusions:**

Interventions targeting associated factors will change global emergency department encounters, although the mutability varies. Policy interventions to address predisposing factors such as substance abuse or access to mental health treatment as well as interventions that speak to enabling factors such as promoting the resiliency of social networks may result in decreased frequency of emergency department utilization.

## Introduction

### Context for Frequent Utilization

Estimates are that U.S. emergency departments collectively experience 130 million encounters annually indicating nearly 43 visits per 100 persons, although the percent of the population that has an emergency department visit each year is less due to the occurrence of frequent and repeated use by a portion of the population [1]. Emergency departments are mandated to provide a screening exam and stabilization to all patients who present [2]. Emergency departments tend to serve a stable population and relatively few emergency department encounters are from new patients [3]. While a small portion of the population seeks the services of the emergency department on a regular basis, frequent emergency department utilization has been associated with demographic variables such as race [4], gender [5], employment status [6], and age [7,8].

Patients with cognitive impairments also have been noted for frequent utilization [9] as are patients with psychiatric conditions [10–15], psychological distress [16–18], and depression [19–22]. This utilization may stem, in part, from the higher prevalence of psychiatric disorders among lower income groups with limited access to mental health care treatment [23]. Patient depressive symptoms also contribute to increases emergency department utilization as suicidality and deliberate self-harm are both common among presentments [17,22,24–28]. Thus, case management for psychosocial problems, homelessness, and substance abuse among frequent users may decrease emergency department use while improving patient care [29–32].

It also has been documented that homeless persons are frequent users of emergency department services [33,34]. This may arise from elevated rates of mental health problems, substance abuse, victimization and traumatic injuries [35–37], lack of transportation, and poor access to primary care [38–40]. These factors contribute to repeated use as the homeless may present to the emergency department with recurring medical conditions that may be managed through community-based services [41,42].

Patient satisfaction with previous emergency department visits has also been associated with return visits [43–45]. Dissatisfaction with a primary care source has been correlated with emergency department presentments for non-urgent conditions [46]. In contrast, those patients that seek the advice of a health professional prior to visiting the emergency department or receive patient-centered care are less likely to frequent the emergency department [47–49].

Self-perceived fair or poor health is often associated with increased utilization [50–52]. Overweight and obese patients have a strong association with the occurrence of chronic medical conditions, reduced health-related quality of life, and increased health care and medication spending. Overweight and obese patients are also more likely to visit the emergency department [53–58]. Given the high prevalence of obesity, clearly elevated disease risks, and increased use of health services, there is great potential for a reduction in health care utilization through efforts in weight reduction and prevention of weight gain [59].

Those insured by Medicaid are more likely to be frequent emergency department users relative to those not covered by Medicaid [22] and, in some cases, those with access to other primary care settings may be more likely to utilize the emergency department relative to those that have limited access [60–62]. The assessment of frequency may need to consider the social construct of medical need [63,64], access [65], and other ambulatory healthcare visits [66,67]. Efforts to address frequent utilization may depend on improving delivery of outpatient care [68–71] and management of chronic conditions such as diabetes and asthma, which are also associated with high levels of emergency department use [4,72].

### Approach

We gather insights through interviews with patients presenting to a regional urban emergency department to examine predisposing, enabling, and need-related factors that are theorized to be associated with frequent utilization of emergency department services. We employ the Andersen-Aday behavioral health model to evaluate the role of these factors relative to the likelihood of encounters with the emergency department [40,73].

### Knowledge Gaps

This study addresses two gaps identified in previous studies. First, since the publication of the initial Andersen-Aday model, later authors, including Andersen, have recommended the extension of the model to include social networks and service quality as predictors of frequent utilization [74]. We address this gap by presenting the conceptualization, measurement, and testing of social networks and service quality variables relative to utilization. Second, within the literature there is a lack of consensus on the particular number of emergency department visits that may characterize frequent utilization. We address this gap by demonstrating that predisposing, enabling, and need-related factors may (or may not) be associated with frequent utilization depending on the a-priori selection of the number of visits that constitute the ‘threshold’ by which frequent utilization is defined.

## Methods

### Study Population and Setting

The study population is derived from a representative randomized sampling (using sampling intervals) of adult patients (18+ years of age) presenting to a level 1 trauma urban hospital’s emergency department (volume 62,000/ppy). The study population consists of patients self-presenting or arriving via Emergency Medical Services (EMS) and triaged using the Emergency Severity Index (ESI) assigned either level 4 or level 5, the least urgent/non-emergent classifications [75,76]. Sampling took place 24 hours, seven days a week over a period of approximately 8 weeks with an 89 percent response rate. Since emergency department presentments are seasonal-cyclical in nature, using historical data we projected the expected volume of patient flow for each day, AM and PM. Using these historical data and estimated response rates, we established a sampling interval for each working shift within a 24 hour period. As the study progressed, we periodically checked the evolving representativeness by cross referencing both the financial and descriptive characteristics of the study population relative to the historical data. Written consent was obtained from all study participants. The study design was vetted and approved by the Institutional Review Board of EVMS (#07-08-EX-0198). Descriptive statistics, including frequencies and averages, are offered for the variables. Relationships with the outcome variable, utilization, are tested using logistic regression to establish odds-ratios.

### Conceptual Framework

The initial Andersen behavioral health model proposed factors that are predictive of healthcare utilization within three dimensions: predisposing characteristics, enabling characteristics, and need characteristics [73]. According to the behavioral health model, there is a loose causal ordering to the three primary dimensions explaining utilization. The predisposing dimension, which is most distant from utilization, embodies many health behaviors that may be considered stressors, predisposing the individual towards medical attention at some point [77]. Predisposing factors are those that are liable to place the individual at risk and in a predisposition to utilize the emergency department. Predisposing characteristics include substance abuse, obesity, and health-belief variables such as knowledge and values. This is followed by enabling factors that facilitate information and access to treatment, such as employment, income, insurance, and access to non-ED primary care doctors. The most proximate dimension to utilization is that of need. Need characteristics include variables related to personal assessments of health or health status.

The Andersen-Aday healthcare utilization model has been adapted to address health utilization among specific populations such as released prisoners with HIV [78], marginally housed [33,79], underserved populations [80], insured populations [68,81], and minority populations [82,83], among others [49,84,85].

Our research uses the Andersen-Aday behavioral health model as the guide to select and organize factors we expect to explain emergency department *frequent* utilization for non-emergent presentments. In a later visitation of the behavioral health model, Andersen expresses the need to incorporate the additional roles of social networks, mental dysfunction, and consumer satisfaction within the model [74]. First, social relationships and networks are viewed as capital that may moderate utilization. The decision to seek the services of a health professional and present at a treatment venue, such as the emergency department, may be made within the context of an individual’s social and familial network. These networks provide access to information and resources that may be used to better manage the condition [86]. Second, mental health needs have been associated with complexity in the treatment of other conditions such as substance abuse and chronic disease [87–89]. Third, Andersen and others encourage the study of the role of consumer satisfaction in promoting utilization [44,74,90,91]. For example, the perceptions of quality of care received have been associated with a willingness to return to the treatment venue to seek additional service [45].

In response to Andersen’s call, we have incorporated within this study a measure of the individual social and familial network, measure of mental health drugs and metal health days as indicators of the psychological disposition, and measure of patients’ perceptions of service quality.

### Emergency Department Utilization

The outcome variable is the self-reported number of emergency department encounters. Patients were asked to estimate the number of additional times they have gone to an emergency department to receive treatment for themselves within the past year. Noting that a patient’s timeframe recall may affect accuracy [92], selected responses were checked with some difficulty against accessible medical records to confirm agreement. The responses were folded into the several possible outcomes of two or more visits, three or more visits, four or more visits, and five or more visits within the past 12 months. Identification of a particular number of emergency department visits that may constitute a threshold or cutoff at which point the presentment may be characterized as *frequent* utilization is not well established [81,93–96]. By way of comparing multiple levels of utilization ranging from two or more encounters through five or more encounters we are able to distinguish differences in likelihoods among different population characteristics organized within the Andersen-Aday framework [67,97].

### Independent Variables

#### Demographic Characteristics

Several demographic variables are examined including Age (<21, 21–30, 31–40, 41–50, 51–60, >60), Gender (male, female), and Race/Ethnicity (white, African American, Hispanic/Latino, Asian/Pacific Islander). Participants self-identified these characteristics.

#### Predisposing Factors

Predisposing factors are those that indicate a predisposition the need to use health services. Participants were queried on being prescribed drugs for mental health issues as well as whether, within the past 30 days, poor mental health kept them from doing usual activities. In addition, prescription drug abuse includes recently taking prescription drugs such as pain killers or stimulants that were either not prescribed or not as prescribed. Further, participants were asked if they had done any drugs such as meth, crack, heroine, or marijuana within the past 24 hours. Gathered also are indicators of whether, within the past 30 days, alcohol use kept the participant from doing usual activities as well as frequency with which six or more drinks are consumed on one occasion. Lastly, participants were assigned ‘at risk’ due to being in either the obese or the overweight BMI CDC categories.

#### Enabling Factors

Enabling factors are those that, when in place, facilitate the propensity to use health services. These include availability and access to health services as well as knowledge about the service. Both employment and insurance status are assessed. Further, participants were queried if they consulted with a health professional about their presenting condition soon before their decision to visit the emergency department. In addition, participants were asked if they spoke with a family member or friend about their presenting condition soon before their decision to visit the emergency department. Patients were assessed whether or not there was a time lag of more than 10 hours between the time that they realized they needed the attention of a medical professional and arrival at the emergency department. Also, respondents were queried about their efforts to make an appointment to see a doctor or nurse prior to their arrival at the emergency department. Lastly, the primary reason for choosing to seek the services of the emergency department, rather than some other healthcare treatment venue, was assessed; measured were participants’ reasons related to the quality of reputation, facilities, personnel, and services.

#### Need Factors

Need factors are those that relate to health and functional status of the patient as well as the patient’s perception that the condition warrants professional attention. Participants were prompted to report the seriousness of their presenting condition as not serious, somewhat serious, or very serious as well as to report the seriousness of their presenting condition on a scale from one to ten, with ten being serious and one being not serious at all. Further, participants were asked if their most recent visit to the emergency department was for the same presenting condition. Lastly, participants were queried on the number of times they were admitted to the hospital within the past 12 months.

### Analysis

We analyze the associations of these factors with the outcome variable, emergency department utilization. We perform logistic regression to identify those factors that are statistically related with the four levels of dichotomous emergency department utilization (two or more visits, three or more visits, four or more visits, and five or more visits), reporting the confidence intervals and adjusted odds ratio (AOR) at each level of utilization. The treatment of the utilization variable as dichotomous, rather than continuous, within these several analyses is justified within the context of the research question which seeks to identify the threshold at which point a particular utilization factor may become significant. Relationships are adjusted for patient characteristics and inflation due to multiple covariates with the Bonferroni adjustment. Covariates are ranked and entered according to explanatory power. Residuals are checked for poor fit including Cook’s Distance residual and Leverage residual. R-square presented for each regression includes Cox & Snell and Nagelkerke. The Roa’s/Wald statistic is checked for significance and Model Chi goodness tests (p < .01) are reported. When reporting the log-odds in the single table it is acknowledge that cases included within the first level of utilization (2 or more visits) will necessarily also be included within the subsequent levels of utilization (e.g., 3 or more visits). Although odds ratios appear within a single table, figures represent separate analyses and each level of utilization is a free-standing analysis. When reporting, caution has been taken not to compare across analyses.

## Results

All 1,443 patients presenting at the emergency department and enrolled in the study have at least a single visit (the current visit), 70.62 percent (1,019 patients) have two or more, 48.3 percent (697 patients) have three or more, 25.36 percent (366 patients) have four or more, and 14.97 percent (216) have five or more emergency department visits within the previous 12 months. Table 1 reports the mean age for the study population (34.4 years, standard deviation 13.4 years), the percent female (62.4 percent), and the percent African American (75.0 percent).

**Table 1:** Demographic Characteristics.

	Participants		2+ Visits		3+ Visits		4+ Visits		5+ Visits	
Characteristic	(n = 1443)		(n = 1019)		(n = 697)		(n = 366)		(n = 216)	
**Age, y, mean (SD)**	34.4	(13.4)	34.0	(13.4)	34.1	(13.3)	34.1	(13.3)	33.8	(13.3)
**Age, y, no. (%)**										
<21	190	(13.2)	138	(13.5)	96	(13.8)	50	(13.7)	33	(15.3)
21–30	528	(36.6)	381	(37.4)	255	(36.6)	133	(36.3)	77	(35.6)
31–40	258	(17.9)	177	(17.4)	124	(17.8)	70	(19.1)	36	(16.7)
41–50	265	(18.4)	185	(18.2)	127	(18.2)	61	(16.7)	37	(17.1)
51–60	140	(9.7)	97	(9.5)	68	(9.8)	38	(10.4)	27	(12.5)
>60	62	(4.3)	41	(4.0)	27	(3.9)	14	(3.8)	6	(2.8)
**Gender, no. (%)**										
Female	901	(62.4)	674	(66.1)**	468	(67.1)*	258	(70.5)***	151	(69.9)**
Male	342	(37.6)	345	(33.9)	229	(32.9)	108	(29.5)	65	(30.1)
**Race/Ethnicity, no. (%)**										
African American	1082	(75.0)	803	(78.8)***	563	(80.8)***	288	(78.7)	172	(79.6)
White	319	(22.1)	200	(19.6)	123	(17.6)	71	(19.4)	40	(18.5)
Hispanic/Latino	19	(1.3)	10	(1.0)	6	(0.9)	3	(0.8)	0.0	(0.0)
Asian/Pac Islander	14	(1.0)	3	(0.3)	2	(0.3)	1	(0.3)	3	(0.5)
Other	9	(0.6)	3	(0.3)	3	(0.4)	3	(0.8)	3	(1.4)

*Note*. Description of above variables presented in text.

Sig.:

*P = .05

**P = .01

***P = .001

### Predisposing Factors

Predispositions to utilize healthcare services include indicators presented in Table 2. Just over 11 percent of all patients presenting at the emergency department with low acuity conditions used prescribed mental health drugs with the past year. As the frequency of emergency department visits increases to 5 or more visits within the past year, so too does the proportion of patients with prescribed mental health drugs, from 11.3 to 18.5 percent. Likewise, nearly 20 percent of all participants report that within the past 30 days poor metal health kept them from doing usual activities; this increases with frequency of utilization to 31.5 percent for those with five or more visits.

**Table 2:** Predisposing Factors.

	Participants		2+ Visits		3+ Visits		4+ Visits		5+ Visits	
	(n = 1443)		(n = 1019)		(n = 697)		(n = 366)		(n = 216)	
**Mental drugs, no. (%)**										
Yes	162	(11.3)	121	(11.9)	100	(14.4)***	58	(15.9)***	40	(18.5)***
No	1273	(88.7)	895	(88.1)	595	(85.6)	306	(84.1)	175	(81.0)
**Mental days, no. (%)**										
Yes	289	(20.1)	231	(22.7)***	185	(26.6)***	104	(28.4)***	68	(31.5)***
None	1147	(79.9)	787	(77.3)	511	(73.4)	262	(71.6)	148	(68.5)
**Prescrp. abuse, no. (%)**										
Yes	136	(9.5)	108	(10.6)*	92	(13.2)***	46	(12.6)*	32	(14.8)***
No	1301	(90.5	908	(89.4)	603	(86.8)	319	(87.2)	183	(84.7)
**Cocaine etc., no. (%)**										
Yes	58	(4.0)	44	(4.3)	29	(4.2)	13	(3.6)	8	(3.7)
No	1378	(95.5)	972	(95.7)	666	(95.8)	352	(96.2)	207	(96.3)
**Alcohol days, no. (%)**										
Yes	39	(2.7)	29	(2.8)	25	(3.6)	13	(3.7)	6	(2.8)
No	1400	(97.3)	989	(97.2)	671	(96.4)	352	(96.3)	210	(97.2)
**Six drinks, no. (%)**										
Some	255	(17.7)	171	(16.8)	112	(16.1)	59	(16.1)	34	(15.7)
Never	1188	(82.3)	848	(83.2)	585	(83.9)	307	(83.9)	182	(84.3)
**BMI CDC, score (SD)**	28.9	(7.5)	29.0	(7.8)	29.2	(8.0)	29.2	(8.4)	29.3	(8.0)
**BMI risk, no. (%)**										
Yes risk	189	(35.2)	139	(38.1)	104	(41.4)	58	(46.4)	31	(42.5)
No risk	348	(64.8)	226	(61.9)	147	(58.6)	67	(53.6)	42	(57.5)

*Note*. Description of above variables presented in text.

Sig.:

*P = .05

***P = .001

Over 9 percent of all participants report recently taking prescription drugs such as pain killers or stimulants that were either not prescribed or not as prescribed; this increases with frequency of utilization to 14.8 percent for those with five or more visit. Approximately 4 percent of participants across all levels of utilization report taking drugs such as meth, crack, heroine, or marijuana within the past 24 hours, 3 percent of participants across all levels of utilization report that, within the past 30 days, alcohol use kept them from doing usual activities, and 16.5 percent of participants across all levels of utilization report the consumption with some frequency of six or more drinks at one occasion.

The mean BMI CDC score for participants is 28.9 (standard deviation 7.5 units). This BMI score increases slightly as emergency department encounters become more frequent. Over 35 percent of participants are ‘at risk’ due to being either overweight or obese and this proportion is as high as 46.4 percent, depending on the level of utilization.

### Enabling Factors

Enumerated in Table 3 are the enabling factors that are expected to enhance the propensity to utilize the emergency department. The percent reporting not being currently employed increases across frequency of utilization from 40 to 53.2 percent. In contrast, the full time employed participants constitute roughly 43 percent of study participants and this proportion decreases with frequency of utilization.

**Table 3:** Enabling Factors.

	Participants		2+ Visits		3+ Visits		4+ Visits		5+ Visits	
	(n = 1443)		(n = 1019)		(n = 697)		(n = 366)		(n = 216)	
**Employment, no. (%)**										
Full time	623	(43.2)	415	(40.7)	265	(38.0)	114	(31.1)**	60	(27.8)*
Part time	243	(16.8)	177	(17.4)	120	(17.2)	69	(18.9)	41	(19.0)
Not employed	577	(40.0)	427	(41.9)	312	(44.8)	183	(50.0)	115	(53.2)
**Insurance, no (%)**										
Medicaid or Medicare	158	(10.9)	124	(12.2)***	99	(14.2)***	64	(17.5)***	44	(20.4)***
Commercial	691	(47.9)	484	(47.5)	325	(46.6)	175	(47.8)	100	(46.3)
Other inc. military	42	(2.9)	30	(2.9)	19	(2.7)	8	(2.2)	7	(3.2)
Uninsured	552	(38.3)	381	(37.4)	254	(36.4)	119	(32.5)	65	(30.1)
**Consult prof., no. (%)**										
No	1069	(74.1)	747	(73.3)	510	(73.2)	251	(68.6)	150	(69.4)
Yes	374	(25.9)	272	(26.7)	187	(26.8)	115	(31.4)	66	(30.6)
**Consult f/f, no. (%)**										
No	271	(18.8)	204	(20.0)**	141	(20.2)	82	(22.4)	45	(20.8)
Yes	1172	(81.2)	815	(80.0)	556	(79.8)	284	(77.6)	171	(79.2)
**Delay, no. (%)**										
No	675	(46.8)	466	(45.7)	322	(46.2)	167	(45.6)	99	(45.8)
Yes	768	(53.2)	553	(54.3)	375	(53.8)	199	(54.4)	117	(54.2)
**Try make apt., no. (%)**										
No	1097	(76.0)	757	(74.3)	514	(73.7)	254	(69.4)	145	(67.1)
Yes	346	(24.0)	262	(25.7)	183	(26.3)	112	(30.6)	71	(32.9)
**Service quality, no. (%)**										
No	1095	(75.9)	763	(74.9)	518	(74.3)	257	(70.2)	152	(70.4)
Yes	348	(24.1)	256	(25.1)	179	(25.7)	109	(29.8)**	64	(29.6)*

*Note*. Description of above variables presented in text.

Sig.:

*P = .05

**P = .01

***P = .001

The uninsured represent just over 38 percent of the study’s emergency department presentments. As frequency of utilization increases, the proportion of the uninsured decrease to 30.1 percent. This is in contrast with those participants with Medicaid or Medicare, where the percent increases from 10.9 to over 20 percent.

Social networks and relationships may be related to health services as posited by Andersen [22]. However, whether social networks and relationships either facilitate or frustrate emergency department utilization is not clear. Compelling theoretical argument could be made that those who have social and familial networks may be more likely to consult with others to gain insights into remedies, self-treatment, and management of the condition as well as receive empathy and compassion. This would suggest that those with networks would be less likely to present at the emergency department for non-emergent conditions relative to those with less social capital. We find that nearly 26 percent of participants consulted a health professional about their presenting condition soon before their decision to visit the emergency department and this increased to over 30 percent with frequency of utilization. Across frequency of utilization, between roughly 77 and 81 percent spoke with a family member or friend about their presenting condition soon before their decision to visit the emergency department.

Roughly 53 percent of respondents report a delay time lag of more than 10 hours between the time that they realized they needed the attention of a medical professional and arrival at the emergency department and this changes little with frequency of utilization. However, 24 percent of participants report trying to schedule an appointment with a primary care professional prior to presenting at the emergency department and this increases steadily with frequency of utilization to 32.9 percent.

As noted above, Andersen (1995) encourages the study of satisfaction with previous health care service encounters and, by extension, suggests that the perception of quality of services received may promote further utilization of the service. In fact, 24.1 percent of participants report that the quality of the emergency department (based upon either reputation, facilities, personnel, or services) was the primary reason for presenting at the emergency department rather than another venue and this increases steadily with frequency of utilization to nearly 30 percent.

### Need Factors

Need factors reflect the status of the patient’s condition and the belief that the condition ought to receive professional attention. As shown in Table 4, the mean score assigned by participants–all of whom were triaged as low acuity, non-emergent–is 7.1 on a ten point range (standard deviation 2.6 points), with little variation across frequency of utilization. Interestingly, while the mean score tends to be high, nearly 10 percent report that their condition is ‘not serious at all’ and about 27 percent report ‘somewhat serious.’ The percent of all participants that identify their presenting condition as ‘very serious’ is 63.4 percent and this increases steadily with frequency of unitization to roughly 74 percent.

**Table 4:** Need Factors.

	Participants		2+ Visits		3+ Visits		4+ Visits		5+ Visits	
	(n = 1443)		(n = 1019)		(n = 697)		(n = 366)		(n = 216)	
**Seriousness, mean (SD)**	7.1	(2.6)	7.3	(2.3)**	7.4	(2.2)	7.6	(2.1)**	7.5	(2.2)
**Seriousness, no. (%)**										
Very Serious	910	(63.4)	692	(68.2)***	436	(69.8)***	271	(74.0)***	158	(73.1)***
Somewhat serious	390	(27.2)	240	(23.6)	162	(23.3)	76	(20.8)	42	(19.4)
Not serious at all	136	(9.5)	83	(8.2)	48	(6.9)	19	(5.2)	16	(7.4)
**Last visit same, no. (%)**										
Yes	356	(25.2)	306	(30.1)***	242	(34.9)***	147	(40.3)***	90	(41.7)***
No	1055	(74.8)	709	(69.9)	452	(65.1)	218	(59.7)	126	(58.3)
**Times admitted, no. (%)**										
None	1152	(79.8)	754	(74.0)***	485	(69.6)***	229	(62.6)***	127	(58.8)***
1 time	200	(13.9)	200	(19.6)	147	(21.1)	80	(21.9)	46	(21.3)
2 times	31	(2.1)	31	(3.0)	31	(4.4)	23	(6.3)	16	(7.4)
3 or more times	34	(2.4)	34	(3.0)	34	(4.9)	34	(9.3)	27	(12.5)

*Note*. Description of above variables presented in text.

Sig.:

**P = .01

***P = .001

Further, just over 25 percent of respondents report that their most recent visit to the emergency department was for the same presenting condition and this proportion dramatically increases to 41.7 percent for those with five or more visits, suggesting frequent visitation for a recurrent condition. Lastly, a sizable majority (79.8 percent) of participants report not having been admitted to the hospital within the past 12 months. Although this decreases with frequency of utilization, remarkably 58.8 percent of those reporting five or more emergency department presentments within the past 12 months also report no hospital admissions, suggesting ED visitation related to the management of a recurrent condition(s).

## Significant Utilization Factors

While recognizing that multiple factors are associated with emergency department utilization, a central objective of this research is not only to test factors that fall within the Andersen-Aday model of predisposing, enabling, and need-based factors, but also to demonstrate that these factors may distinguish themselves when examined at different levels of utilization. Table 5 summarizes those factors appearing in the previous four tables that demonstrate a significant relationship at one or more levels of utilization. After adjusting for covariates including demographic factors, we report the odds ratios for these relationships.

**Table 5:** Significant Factors Associated with Four Levels of ED Utilization.

	2+ ED Visits			3+ ED Visits			4+ ED Visits			5+ ED Visits		
	Odds	95%		Odds	95%		Odds	95%		Odds	95%	
Characteristics	Ratio	C.I.	*P*	Ratio	C.I.	*P*	Ratio	C.I.	*P*	Ratio	C.I.	*P*
**Demographic**												
Gender (Female)	1.75	1.13,2.71	.01	1.64	1.08,2.49	.02	2.36	1.39,4.03	.00	1.48	1.08,2.02	.01
Race (Black)	2.10	1.29,3.42	.00	2.36	1.45,3.89	.00	*1*.*27*	*0*.*71*,*2*.*28*	.*42*	*1*.*51*	*0*.*92*,*3*.*31*	.*26*
**Predisposing**												
Mental Drugs (Yes)	*1*.*24*	*0*.*85*,*1*.*83*	.*26*	1.85	1.32,2.60	.00	1.76	1.25,2.49	.00	2.06	1.39,3.04	.00
Mental Days (Yes)	1.90	1.37,2.63	.00	2.26	1.73,2.97	.00	1.91	1.46,2.52	.00	2.80	1.51,2.88	.00
Prescrp. Abuse (Yes)	1.67	1.08,2.59	.02	2.43	1.66,3.56	.00	1.56	1.07,2.28	.02	1.87	1.22,2.86	.00
**Enabling**												
Medicaid/Care (Yes)	1.79	1.17,2.75	.00	2.06	1.45,2.93	.00	2.30	1.63,3.25	.00	2.57	1.75,3.77	.00
Consulted F/F (No)	2.09	1.12,3.82	.01	*1*.*44*	*0*.*87*,*2*.*38*	.*16*	*1*.*49*	*0*.*84*,*2*.*68*	.*17*	*1*.*32*	*0*.*66*,*2*.*64*	.*43*
Employed FT (No)	1.49	1.16,1.88	.00	1.55	1.25,1.92	.00	2.02	1.57,2.60	.00	2.24	1.68,3.08	.00
Serv. Quality (Yes)	*1*.*17*	*0*.*88*,*1*.*54*	.*27*	*1*.*16*	*0*.*91*,*1*.*47*	.*25*	1.47	1.12,1.92	.01	1.38	1.01,1.90	.05
**Need**												
Seriousness (Unit)	1.14	1.02,1.22	.01	1.09	1.01,1.19	.05	1.17	1.05,1.30	.00	*1*.*11*	*0*.*98*,*1*.*27*	.*09*
Very Serious (Yes)	2.02	1.59,2.57	.00	1.72	1.38,2.14	.00	1.91	1.47,2.49	.00	1.68	1.22,2.33	.00
Last Visit (Yes)	4.09	2.24,7.47	.00	2.54	1.61,4.01	.00	2.59	1.59,4.24	.00	2.71	1.52,4.82	.00
Admitted (Yes)	5.53	3.99,7.58	.00	5.43	3.94,7.51	.00	4.27	3.23,5.66	.00	4.05	2.96,5.55	.00
	R^2^ = .20^1^, .29^2^			R^2^ = .19^1^, .25^2^			R^2^ = .17^1^, .22^2^			R^2^ = .15^1^, .20^2^		
	Model χ^2^(21) = 301, p < .01			Model χ^2^(21) = 275, p < .01			Model χ^2^(21) = 216, p < .01			Model χ^2^(21) = 152, p < .01		

Italicized figures represent associations that do *not* meet significance at *P* = .05, *P* = .01, or *P* = .001 levels.

^1^Cox & Snell

^2^Nagelkerke

For example, within demographic characteristics, the reported odds ratio represents the ratio between the odds of two or more emergency department visits by females over the odds of two or more visits by males. The odds are 1.75 to 1 that females will have two or more visits relative to males, meaning that females are .75 times more likely to have two or more visits relative to males (sig = .01). Females are statistically more likely, relative to males, to be frequent utilizers across all four levels of utilization. Further, Black Americans are more than twice as likely to report two or more and three or more emergency department visits relative to non-Black Americans (2.10 OR sig = .00 and 2.36 OR sig = .00, respectively). However, we do not find a statistical difference in the utilization odds for either four or more or five or more visits.

### Predisposing Factors

We find evidence that the usage of prescription drugs for mental health issues is associated with utilization. For example, those patients reporting mental health drugs are twice as likely (2.06 OR sig = .00) to utilize the emergency department five or more times relative to those patients that do not use prescription mental health drugs. Notable, though, users of prescription mental health drugs do not appear to be statistically more likely to have two or more visits, suggesting a threshold effect. We also find evidence that, across all four levels of utilization, patients who report that poor mental health kept them from doing usual activities are more likely to utilize the emergency department relative to those that do not report poor mental health, from .90 times more likely (sig = .00) for those with two or more visits to 1.80 times more likely (sig = .00) for those with five or more visits. The taking of prescription drugs that were not prescribed is associated with frequent emergency department utilization among all levels of utilization from .67 times more likely (sig = .02) for those with four or more visits to .87 times more likely (sig = .00) for those with three or more visits.

### Enabling Factors

Medicaid/Medicare patients are strongly associated with frequent utilization, from .79 times more likely (sig = .00) for those with two or more visits to 1.57 times more likely (sig = .00) for those with five or more visits. Notable, though, the characteristic of uninsured is not presented within this table, meaning that the odds ratio for the uninsured vs the insured is not significant at any of the utilization thresholds suggesting that the uninsured are statistically no more likely to be frequent utilizers relative to the insured.

We find evidence in two key areas of investigation encouraged by Andersen (1995), social networks and service quality. First, we find that those patients that did not consult with a friend or family member about their condition just prior to presentment at the emergency department are twice as likely (2.09 OR sig = .01) to report two or more visits relative to those that consulted a friend or family member. This suggests that consulting with a friend of family member decrease the likelihood of frequent utilization. Second, we find evidence that perception of quality services is associated with utilization of the emergency department. Those patients that have an enhanced perception of the quality of services delivered at the emergency department are .47 and .38 times more likely (sig = .01 and sig = .05, respectively) to report four or more and five or more visits.

Those that are not employed full time, relative those employed full time, are associated with utilization, increasing across all levels from nearly 50 percent more likely (1.49 OR sig = .00) for those reporting two or more visits to over twice as likely (2.24 OR sig = .00) for those reporting five or more visits.

### Need Factors

Multiple need-related factors are found associated with emergency department utilization. Seriousness of presenting condition ranges from one to ten and the expectation is that those reporting a relatively high seriousness score for their presenting condition will also be more frequent utilizers of the emergency department. For example, we find that a one unit increase in seriousness results in a .14 change in the log-odds (sig = .01) for patients reporting two or more visits. In addition, across the thresholds, those patients that characterized their condition as ‘very serious’ are more likely to report frequent visits relative to those that report their condition as not serious or somewhat serious ranging from .68 to 1.02 times more likely (sig = .00).

The data also confirms that, across all levels of utilization, patients reporting that the most recent visit to the emergency department was for the same presenting condition are more frequent utilizers relative to those that do not present with a condition similar with their last visit. Last, those that report multiple encounters with the emergency department are between 3.05 and 4.53 times more likely to have been admitted to the hospital as not admitted to the hospital (sig = .00).

## Discussion

### Gaps Addressed

The intent of this research is to address two gaps. As noted above, there is a gap within the literature on what threshold may constitute *frequent* utilization of healthcare services, including emergency department visitation for non-emergent and primary care needs. Our approach does not presuppose that there is a universal discrete number of visits that constitute frequent utilization. Rather, a range of utilization is established and variation among the covariates is explored to identify at which point the attributes within a covariate no longer have significance. Through this approach we visually illustrate (in Table 5) the point where a covariate no longer has explanatory power. Notable is that half the covariates are significant across all levels of utilization; for these variables the choice of a-priori defining ‘frequent’ utilization in terms of a particular number of encounters will not be of central importance in finding significant relationships. However, for other covariates, the choice of a utilization threshold may determine whether or not the relationship has significance.

Another gap addressed in this study is the measurement and testing of the two enabling factors, social networks and service quality, both of which are explored as natural extensions of the Andersen-Aday behavioral health model. Strong social networks may facilitate information sharing and provide a support network in the management of the condition. Thus, it has been argued, these relationships could moderate an individual’s decision about where to seek professional medical attention. Although our findings are initial, it appears that a support system on which an individual may draw social capital may be associated with fewer encounters with the emergency department. Further, we find confirmation for the theoretically argued connection with service quality. For example, those that report visiting the emergency department either four or more times are more likely to cite service quality as the primary motivating factor for seeking services at the emergency department rather than elsewhere.

### Summary of Findings

We find that repeat visits to the emergency department within a 12 month period are quite common: over 70 percent have visited an emergency department at least one other time and nearly 15 percent of low acuity/non-emergent patients have five or more emergency department visits. Among our demographic variables, frequency of utilization does not vary much by age, but females are more likely relative to males to have frequent utilization across all encounter levels.

We find evidence for predisposing factors: both poor mental health and mental health-related prescription medications signify frequent utilization. In addition, nearly 20 percent of participants report poor metal health keeping them from doing usual activities and this proportion increases markedly with frequency of utilization.

Although substance abuse has been theorized to be related to utilization, we have mixed findings. The abuse of prescription drugs is clearly associated with increased frequency of utilization. Yet, on the other hand, we find that taking drugs such as meth, crack, heroine, or marijuana within the past 24 hours, alcohol keeping one from usual activities, and consumption with some frequency of six or more drinks are not associated with more frequent utilization.

Enabling factors are also found to be associated with utilization. The proportion of patients that are either not employed or part-time employed increases with frequency of utilization. We find that those that are not employed full time are more than twice as likely to report five or more visits. In an effort to better understand this we performed additional analyses (not presented here) to see if there are interaction effects with insurance, time of presentment, or inability to make an appointment with a primary care professional (all of which did not significantly alter our findings here).

Further, the odds ratios for the uninsured relative the insured is not significant suggesting that both the uninsured are statistically no more likely to be frequent utilizers relative to the insured and insurance status is not a good discriminator of utilization frequency. In contrast, Medicaid/Medicare patients are several times more likely to be high frequency utilizers relative to the rest of the population. This suggests that these programs, intended to facilitate access, are correlated with frequent encounters. Last, consistent with Andersen’s view of the primacy of need in explaining utilization, all the need factors within this research demonstrate significance across all the levels of utilization.

### Limitations

Several of these variables are global measures and the presentments are not particular to an ailment, disease, or condition [45]; thus, the findings may not inform clinical practice settings [98]. Nonetheless, these findings may inform broader healthcare policy as Andersen notes the mutable nature of model factors, especially those that enable healthcare access [74].

Further, the focus is on a single emergency department within a region with a sizable transitional population and several hospital systems making validation of self-reported frequency through examination of medical records difficult. In addition, these data were collected through interviews of patients while in the process of encountering the emergency department and perceptual questions relating to why the patient sought treatment from the emergency department rather than some other treatment venue are difficult to validate. Last, implicit in the behavioral health model is a causal ordering among the three dimensions, yet in this research we have treated the covariates as independent and examined the main effects and do not offer a systematic exploration of the interactions.

### Conclusion

We applied the Andersen-Aday behavioral health model to organize and assess factors associated with frequency of utilization, including social networks, mental health, and service quality factors. This research strongly suggests that changes in predisposing, enabling, and need-based factors will change global emergency department encounters. However, the mutability of these factors varies. Policy interventions to address predisposing factors such as substance abuse or access to mental health treatment may have returns on investments not only in terms of quality of life, but also overall cost savings to the health system. Interventions that speak to enabling factors such as promoting the resiliency of social networks, especially among more senior populations, may result in decreased emergency department utilization. Among these dimensions, need is the most proximate to utilization. Since both predisposing and enabling factors either enhance or frustrate the probability of need, such interventions may be expected to have a collateral affect upon utilization.
